# Refined movement analysis in the staircase test reveals differential motor deficits in mouse models of stroke

**DOI:** 10.1177/0271678X241254718

**Published:** 2024-05-14

**Authors:** Matej Skrobot, Rafael De Sa, Josefine Walter, Arend Vogt, Raik Paulat, Janet Lips, Larissa Mosch, Susanne Mueller, Sina Dominiak, Robert Sachdev, Philipp Boehm-Sturm, Ulrich Dirnagl, Matthias Endres, Christoph Harms, Nikolaus Wenger

**Affiliations:** 1 Department of Neurology with Experimental Neurology, Charité – Universitätsmedizin Berlin, Berlin, Germany; 2 QUEST Center for Transforming Biomedical Research, Berlin Institute of Health (BIH), Berlin, Germany; 3 Center for Stroke Research Berlin, Charité - Universitätsmedizin Berlin, Berlin, Germany; 4 NeuroCure Cluster of Excellence and Charité Core Facility 7T Experimental MRIs, Charité-Universitätsmedizin Berlin, Berlin, Germany; 5 Institute of Biology, Humboldt University of Berlin, Berlin, Germany; 6 Sussex Neuroscience, School of Life Sciences, University of Sussex, Brighton, UK; 7 DZHK (German Center for Cardiovascular Research), Berlin, Germany; 8 DZNE (German Center for Neurodegenerative Diseases), Berlin, Germany; 9 DZPG (German Center of Mental Health), Berlin, Germany

**Keywords:** Machine learning, motor deficits, rodent models, stroke, translational research

## Abstract

Accurate assessment of post-stroke deficits is crucial in translational research. Recent advances in machine learning offer precise quantification of rodent motor behavior post-stroke, yet detecting lesion-specific upper extremity deficits remains unclear. Employing proximal middle cerebral artery occlusion (MCAO) and cortical photothrombosis (PT) in mice, we assessed post-stroke impairments via the Staircase test. Lesion locations were identified using 7 T-MRI. Machine learning was applied to reconstruct forepaw kinematic trajectories and feature analysis was achieved with *MouseReach*, a new data-processing toolbox. Lesion reconstructions pinpointed ischemic centers in the striatum (MCAO) and sensorimotor cortex (PT). Pellet retrieval alterations were observed, but were unrelated to overall stroke volume. Instead, forepaw slips and relative reaching success correlated with increasing cortical lesion size in both models. Striatal lesion size after MCAO was associated with prolonged reach durations that occurred with delayed symptom onset. Further analysis on the impact of selective serotonin reuptake inhibitors in the PT model revealed no clear treatment effects but replicated strong effect sizes of slips for post-stroke deficit detection. In summary, refined movement analysis unveiled specific deficits in two widely-used mouse stroke models, emphasizing the value of deep behavioral profiling in preclinical stroke research to enhance model validity for clinical translation.

## Introduction

Worldwide, stroke is one of the leading causes of long-term disability.^1^ About three out of four stroke survivors suffer from acute upper limb deficits.^2^ A central objective of translational stroke research is to better understand the prognosis of motor recovery and find personalized treatment strategies.

One important predictor of post-stroke recovery is the severity of the initial deficit.^3–5^ After severe paresis, only half of stroke survivors regain meaningful levels of upper limb function.^6^ The recovery of arm movements benefits to some extent from specialized forms of physiotherapy, such as constraint-induced movement therapy, mental practice, robotics, or EMG biofeedback.^7^ Yet, deficits in fine-skilled hand movements persist across treatment modalities in patients.^7^

Lesion location is a second important factor predicting post-stroke recovery. In humans, isolated cortical stroke displays a higher degree of upper limb recovery compared to subcortical stroke in the corona radiata, basal ganglia, or thalamus.^8^ Different lesion locations are characterized by heterogeneous symptoms in humans. For example, a stroke along the corticospinal tract leads to sudden-onset hemiparesis.^9,10^ In contrast, symptoms after isolated basal ganglia infarct can manifest with prolonged aggravation, for weeks and months, with movement disorders such as dystonia or hyperkinesia.^11^ In rodent stroke models, it is not known whether distinct lesion locations will similarly relate to specific behavioral deficits or follow the same recovery course.

Typical assessments of forelimb function in rodent stroke models capture global behavioral parameters such as pellet retrievals, percentage of limb use in a cylinder, or paw placement on a ladder.^12,13^ These global scores measure changes in performance but lack the capacity to discriminate cognition from sensorimotor function or sickness behavior. When using such global metrics, it is further difficult to distinguish true recovery from the development of compensatory movement strategies after stroke.^14–16^ These limitations have been readily recognized by interdisciplinary expert consortia (STAIR, SRRR), and it has been recommended to integrate kinematic analysis as broadly as possible for the assessment of sensorimotor function in preclinical stroke research.^17,18^

Machine learning has opened compelling avenues for studying complex motor behaviors in rodents without the need for marker-based motion tracking.^19–26^ These tools have helped unravel neural circuits responsible for coordinated forepaw function in physiological states in mice.^27,28^ More recently, these tools have been successfully adopted for the analysis of gait in mice following cortical photothrombosis.^29^ Given these new technological opportunities, deep behavioral profiling^30^ is about to reveal its full potential for improving translational stroke research.

Here, we examined whether machine learning can refine the analysis of skilled forelimb use and potentially distinguish lesion-specific deficits in mice after stroke. We chose to work with the Staircase test, a well-defined environment for studying coordinated movement.^18,31^ In the task, animals enter into a small reaching chamber to then dynamically grasp food pellets that are placed on a staircase at different distances. During reaching attempts, animals maintain their body stability through an isometric grip with their opposite forepaw on an elevated platform. Instead of relying on traditional pellet counts, we reconstructed bilateral forepaw and food pellet trajectories during task execution with the software package *DeepLabCut.*^19^ Next, we developed *MouseReach*, an automated data-processing toolbox, to derive meaningful parameters for the quantification of post-stroke motor performance. Our algorithms automatically detected reaching attempts towards the pellets and vertical slips of the forepaw from the stabilizing platform. Reaching attempts were further classified into reaches without pellet contact, successful pellet removals or pellet drops during retrieval. The toolbox was further used to analyze kinematic features of reaching attempts, yielding a total parameter set of 30 outcome parameters for refined motor deficit quantification. Application of *MouseReach* accurately captured the time course of differential symptom manifestation following a middle-cerebral artery occlusion (MCAO) or cortical photothrombosis (PT) in mice. We further validated *MouseReach* by examining the therapy effect of citalopram - a selective serotonin reuptake inhibitor (SSRI) - in the PT model.

## Methods

### Experimental design

We used 8–10-week-old C57BL/6N (20–26 g) mice from Charles River Laboratories in our experiments. Animals were housed in enriched home cages in groups of three with a regular day-night cycle. A total of 12 mice (6 females and 6 males) were included in the MCAO group and 10 mice (5 females and 5 males) in the PT group for the analysis of post-stroke deficits. One female mouse was later excluded from the PT group due to anesthesia-related death, and two mice were excluded from the MCAO group after not learning the Staircase task prior to the experimental stroke. A subcutaneous chip was implanted to allow for daily measurements of animal identity and body temperature. Three days before the start of the Staircase training, animals received restricted access to food. Food access was provided ad libitum for 3 hours a day, immediately after the Staircase exposure. For the rest of the day-night cycle, an additional 1.1 g of food per animal was added to each home cage. Daily training and weekly functional assessments were performed between 8 and 11 a.m. In the case of weight loss greater than 5% of bodyweight compared to baseline, the amount of food was increased. Food restriction was suspended for weight loss greater than 10%. Water was available ad libitum. In a separate experiment, the treatment effect of SSRI after PT was investigated using mice randomly assigned to the SSRI group (4 female, 3 male) and the saline control group (3 female, 4 males). Two animals per group had to be excluded from the analysis, because they did not learn to reach for any pellets before stroke. Starting on the second day after surgery, the SSRI group received a daily injection of 10 mg/kg citalopram i.p. (ThermoFisher). The saline group received a daily vehicle injection (0.9% NaCl). All experiments conducted in this study received approval from the Berlin State Office for Health and Social Affairs (Landesamt für Gesundheit und Soziales, LAGeSo) under the licenses G0108/20 and G0343/17. The experiments were carried out in strict accordance with the German Animal Welfare Act and were reported in compliance with the Animals in Research: Reporting In Vivo Experiments (ARRIVE 2.0) guidelines.^32^

### Staircase training

Following two weeks of handling, the animals started training in the Staircase for 30 minutes per day to learn how to grab and eat dustless sugar pellets (20 mg sucrose, TestDiet) with the left and right forepaw. After one week, the duration of the reaching session was reduced to 20 minutes. The remaining pellets in each Staircase box were counted after every trial and reported as the ‘traditional pellet count’. Mice that removed less than two of eight available pellets per side were excluded from the experiment prior to the induction of ischemia. Animals participated daily in the Staircase test, except for the day of stroke intervention.

### Stroke models

For all surgeries, mice were kept under 1%–2% isoflurane anesthesia. Analgesia was achieved with an s.c. injection of carprosol (5 mg/kg). Body temperature was maintained at 37 °C on a heating pad. For photothrombosis (PT), mice were transferred to a stereotactic frame (Kopf Instruments) to receive a unilateral ischemic lesion of the left sensorimotor cortex, as previously described.^33^ In brief, the skull was exposed by a midline incision of the scalp. An opaque, reflective template with a defined opening (3 mm wide, 5 mm long) was aligned to the midline over the left sensorimotor forelimb and hindlimb areas: −2 to +3 mm A/P, 0 to −3 mm M/L related to bregma. Five minutes after an intraperitoneal injection of 200 µL of Rose Bengal (10 mg/ml in 0.9% NaCl; Sigma-Aldrich), the skull was illuminated with a light source (Zeiss, CL 1500 HAL, 150 W, 3000 K) that was securely placed on top of the skull for 15 minutes. For MCAO, we used a transient, 45-minute occlusion of the left middle cerebral artery.^34^ Ischemia was induced by a careful dissection of the left common carotid artery and subsequent introduction of a 7–0 silicon rubber-coated MCAO suture with a coating length of 9–10 mm (monofilament 7019910PK5Re, Doccol Corp., Sharon MA, United States) into the left internal carotid artery and advancing it up to the anterior cerebral artery, thereby occluding the left middle cerebral artery (MCA). The filament was later withdrawn after an occlusion time of 45 min. After both procedures, the animals were allowed to recover in a heated cage for a minimum of 1 hour. Following surgery, all animals had access to wet food. If any signs of dehydration occurred, animals were treated with additional subcutaneous saline injections. The successful induction of stroke was confirmed by observing a lesion volume exceeding 2 mm^3^ in T2-weighted MR images in both stroke models.

### 7T-MRI

T2-weighted images were acquired one day after stroke in the MCAO and PT groups. An experienced researcher used ANALYZE software (v5.0, AnalyzeDirect, Overland Park, KS, USA) to segment the hyperintense lesion. MR-images and the lesion mask were registered on the mouse Allen Brain Atlas using the in-house developed MATLAB toolbox ANTx2 (https://github.com/ChariteExpMri/antx2). Incidence maps expressing the percentage of animals with a lesion in a voxel were plotted for each group in atlas space, and edema-corrected lesion volumes were calculated, as previously described.^35^ Brain region-dependent infarct size was defined as the percentage of lesioned isocortex or striatum.

### Multi-Staircase setup

**Figure 1.**
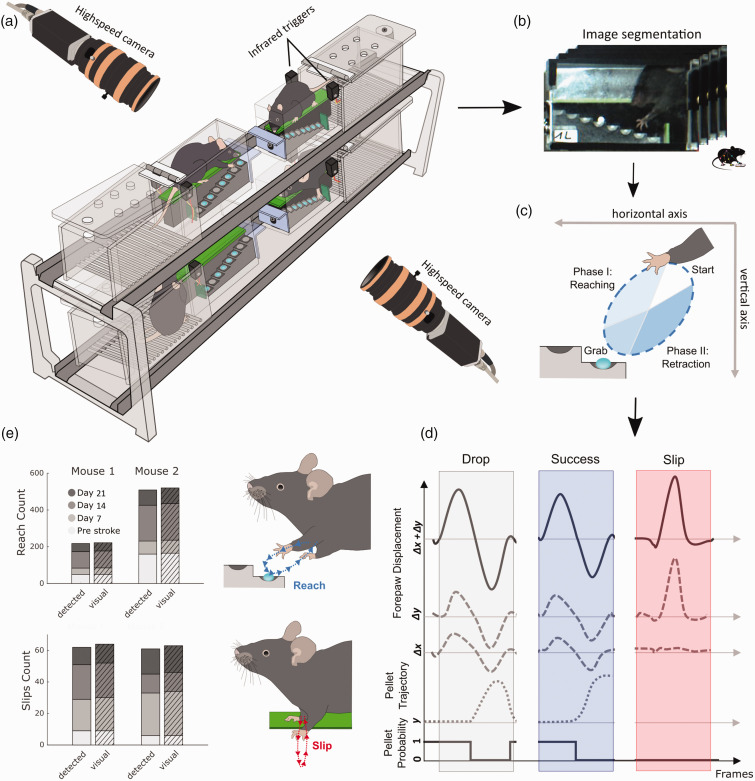
Setup for refined movement analysis and validation of algorithm performance. (a) Four Staircase boxes are simultaneously recorded using two highspeed cameras that are triggered by infrared beams, when mice enter the reaching chambers. (b) Images from individual video-frames are segmented and streamlined for machine learning based motion tracking of forepaw and pellet trajectories in individual Staircase boxes. (c) The direction of forepaw movement during reach cycles defines movement towards (reach-phase) and away from a target sugar pellet (retraction-phase). (d) Successful and unsuccessful reaches are detected based on logical threshold operations for forepaw and pellet displacements. Slips were characterized by sudden vertical movements, independent of pellet information. Probability denotes the probability of pellet existence in a staircase well. Δx and Δy: horizontal and vertical forepaw displacement; y: vertical pellet position. (e) The validation of automatically detected reaches and slips shows high degrees of accuracy in comparison to visual annotation by blinded raters. Data in e present results for two mice from the MCAO and PT groups, before and after stroke on days 7, 14, and 21.

To reduce overall experimental workload for testing multiple animals, we designed a Staircase setup for simultaneous video recordings in four mice (Figures 1 and S1). For this, four Staircase boxes were positioned in a 2 × 2 grid using a customized positioning platform. The platform was designed to guide the accurate placement of each Staircase box, and to minimize time for camera calibrations across days. Each Staircase box consisted of two sub-compartments, a resting chamber, and a reaching chamber containing a dual 8 well staircase (Fig. S1A). We placed one sugar pellet per staircase well to help software algorithms determine when wells were either full or empty. At the entry to the reaching-chamber, each Staircase box was equipped with an infrared break-beam sensor (Adafruit) to detect periods of reaching activity and to prevent overly large video file sizes. Beam break crossings triggered the recording of two high-speed cameras (ACE GigE, Basler, Germany) that were positioned on either side of the Staircase setup. Videos were recorded at a time resolution of 100 frames per second (fps) with a spatial resolution of 640 × 480 pixel. The camera image was centered on four opposing reaching chambers for parallel recordings of multiple mice (Figure 1(a)). In real-time, a customized trigger box (Fig. S1B) integrated information from all beam breaks and performed a logical AND operation to avoid repeated start signals when several animals entered the reaching chambers. Individual recordings stopped when all mice returned to their resting chambers.

### MouseReach software

The toolbox *MouseReach* consists of a pipeline for data-file processing, event classification, and kinematic analysis that generates a set of 30 outcome parameters for each side of the body (Table S1). The toolbox source code is freely available for download from online repositories under the link: https://github.com/Wenger-Lab/MouseReach. Algorithms are both applicable to videos from single and multi-Staircase setups.

#### Data-file processing

Prior to machine learning, we segmented individual videos into four subsections, each showing the image of one of the four reaching chambers (final resolution of 320 × 240 pixel per subsection). Next, we matched video subsections to blinded animal identities from manual entries. The resulting dataset was used to train a neural network with the software package DeepLabCut.^19^ A total of approximately 500 video frames were manually marked as a training set. Video stacks were then automatically streamed to DLC for marker less tracking of forepaw and pellet trajectories in 2D. A custom true-or-false filter discarded non-physiological trajectory jumps that remained present in the DLC neural network despite optimized training. Non-physiological trajectory jumps were defined as deviations of six times the standard deviation for the 2D trajectories. Missing data points that typically affected single video frames were filled with the x and y mean values of the two nearest, correct video frames. All trajectories were then smoothened with a moving mean of 10 frames to reduce trajectory noise.

#### Event classification

Both reaching attempts and forepaw slips were identified based on forepaw velocity when a successive pair of maximal and minimal velocities exceeded an empirically determined threshold (1.5 pixels per frame). The classification of events into either reaching attempts or slips was achieved by calculating the ratio of forepaw displacement in horizontal ( $\bar{\Delta x})$ and vertical ( $\bar{\Delta y})$ directions. A ratio smaller than 1.5 identified slips. Detection of pellet removals was achieved by computing a frame-by-frame binary signal based on the probability of pellet existence in each of the eight staircase wells. A pellet removal was detected when the probability dropped below 99%. The pellet removal was then matched in time with the corresponding reaching attempt. Starting from the frame of pellet removal, a common trajectory of the pellet and the forepaw was monitored. If the pellet disappeared prior to the next reaching attempt and no pellet drop was detected, the reaching attempt was counted as successful (reaches_successful_). Reaching attempts were counted as unsuccessful if a pellet drop was detected during or after a reach. The target for each reaching attempt was calculated by finding the closest staircase well to the inversion point of forepaw movement in 2D. Using this information, we defined a new coefficient of reaching success (k_success_):

$$k_{\mathrm{success}}=\frac{{\mathrm{reaches}}_{\mathrm{successful}}^{2}}{{\mathrm{reaches}}_{\mathrm{all}}}$$

The term *reaches_all_* represents the total count of reaches towards staircase wells, that were not yet emptied by the mouse. *reaches_successful_* counts all successful pellet retrievals from the set of *reaches_all_.* In the formula, we square the term *reaches_successful_* to reward mice with a higher score when they retrieve a higher number of pellets. Together, the event classification algorithms resulted in five outcome parameters of motor performance: success coefficient and number of success events, pellet events, reach events and slip events.

##### Kinematic Analysis

During analysis, pixels and frames were converted to centimeters and seconds, using staircase dimensions from the video and frame rate. Reaching movements were further divided into two discrete phases: a ‘reach’ phase towards or a ‘retraction’ phase away from the pellet (Figure 1(c)). The start, midpoint, and end of a single reaching attempt were calculated based on zero crossings of forepaw velocity. For each of the three categories (full reaching attempt, reach, and retraction phases), we calculated distance, duration, average velocity, and average acceleration. For instantaneous velocity and acceleration, we also determined the respective minima and maxima. For slip events, we calculated ‘slip depth’ as the distance of the vertical forepaw displacement from the green staircase platform (Figure 1(e)). All calculations were performed as Euclidian metrics. For forepaw displacement during reaching (*Δx + Δy*), the total path length ***s*** was calculated as

$$s=\sum_{\mathrm{frame}=1}^{\mathrm{end}}\sqrt{{\Delta x}_{\mathrm{frame}}^{2}+{\Delta y}_{\mathrm{frame}}^{2}}$$

with *frame = 1* and *end* being the starting and end frame of each detected reaching event.

Frame-by-frame speed ***v*** and acceleration ***a*** were quantified as time derivatives:

$$v_{\mathrm{frame}}=\frac{1}{\Delta t}\sqrt{{\Delta x}_{\mathrm{frame}}^{2}+{\Delta y}_{\mathrm{frame}}^{2}}a_{\mathrm{frame}}=\frac{\Delta v}{\Delta t}=\frac{1}{\Delta t^{2}}\sqrt{{\Delta x}_{\mathrm{frame}}^{2}+{\Delta y}_{\mathrm{frame}}^{2}}$$

with Δt being the time interval per video-frame (10 ms at a framerate of 100 Hz).

Together, these calculations resulted in a total of 30 outcome parameters for event quantification and kinematic analysis (Table S1).

## Statistics

The planning of group sizes was performed prior to experiments, based on expected effect sizes for stroke induced changes in pellet count, calculated separately for each stroke intervention (for MCAO: effect size 0.85, alpha error 0.05, resulting group size = 11. For PT: effect size: 0.95, alpha error: 0.05, resulting group size = 9). Experimenters and raters were blind to group allocation. All presented analysis was performed by automated algorithms. To find outcome parameters that maximize the difference between stroke models and timepoints of observation, we used linear discriminant analysis (LDA). Normal distribution was tested with the Kolmogorov-Smirnov test. For statistical quantification of individual outcome parameters, we performed a nonparametric, two-way repeated measures ANOVA for the factors stroke model and timepoint.^36^ For post-hoc analysis, pairwise comparisons were performed using two-tailed Wilcoxon rank sum test (for comparisons between different stroke groups), and the Wilcoxon signed rank test (for paired comparisons within the same stroke group at different timepoints). In the SSRI experiment, we performed pairwise post-hoc group comparisons using a one-tailed test, considering our prior knowledge of the direction of the post-stroke deficit observed in the PT model. Due to the novelty of calculated functional parameters, statistical comparisons are of an exploratory nature, and p-values have not been adjusted for multiple testing. In the figures, all bar graphs are reported using mean ± standard deviation (SD) and box-and-whisker plots are displayed using the Tukey method. Correlations between lesion volume and symptoms were calculated with linear regressions and Pearson correlation coefficients. The accuracy, sensitivity, and specificity of automated event classifications were calculated in comparison to manual annotations as a ground truth. The manual annotation was performed with a custom-programmed graphical user interface in MATLAB by blinded raters.

## Results

### Performance validation of *MouseReach* for movement classification

We first validated the performance of our automated movement classifications in comparison to manual annotations, as gold standard. Automated classification was based on the information of two high-speed video cameras (framerate 100 Hz) that recorded mouse behavior from either side of the multi-Staircase setup (Figure 1(a)). Images of each video frame were then automatically segmented into four individual chambers and streamlined to the machine learning software, *DeepLabCut*, for tracking of forepaw and pellet trajectories (Figure 1(b)). Forepaw trajectories were used to detect reaching attempts and vertical forepaw slips. Reaching attempts were further subdivided into a reaching phase towards and a retraction phase away from the pellet (Figure 1(c)). Targeted pellets were identified by the nearest pellet to the forepaw at the inversion of the two reaching phases. Reaching attempts were further classified into reaches with no pellet contact, successful pellet retrieval or unsuccessful retrieval when the target pellet dropped onto the staircase floor prior to the next reaching event. Sudden downward shifts of the forepaw, limited to the vertical axis, were detected as forepaw slips from the staircase platform (Figure 1(d)).

After establishing the processing algorithms, we validated the performance of automated classification for the event categories: reaching attempts, forepaw slips, pellet removals and final pellet count in the box (Figures 1(e) and S2). As a ground truth, one mouse from each stroke group was randomly picked for manual quantification of all events on four recording days, including one day of pre-stroke behavior. Reaching attempts were correctly identified with an accuracy of 98.3%, a sensitivity of 98%, and a specificity of 100%. Slip detection showed a performance of 99.5%, 96.9%, and 100%, respectively. Pellet removals were identified at 99.8%, 98.5%, and 99.9%. This high degree of accuracy confirmed the suitability of the automated algorithms for the analysis of stroke deficits at group levels. The high number of recorded events (i.e., up to 200 reaching attempts per animal per day) rendered manual annotation very laborious (approx. 4 hours of annotation per animal per recording day), further highlighting the importance of automated processing.

### Quantification of lesion volume and location with MRI morphology

**Figure 2.**
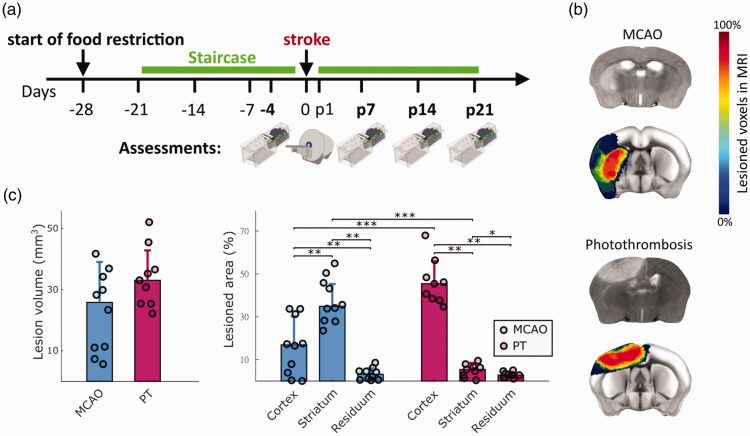
Experimental timeline and quantification of lesion volumes and locations. (a) Animals were trained in the Staircase daily (green bar), except for the day of stroke surgery. Video recordings were performed on day 4 before, and days 7, 14, and 21 after stroke. Days before and after stroke are indicated with symbol ‘-’ and letter ‘p’. MRI was performed on day 1 after stroke. (b) Representative MRI cross sections after MCAO and PT (grey scale images), and colored stroke incidence maps at the group level for MCAO (n = 10) and PT (n = 9) mice. Color bar indicates percentage of mice that showed lesions in individual MRI voxels. (c) Total lesion volume and percentage of lesion affecting cortical, striatal, and residual brain areas. Percentages in c are referenced to the stroke affected hemisphere. Bar graphs are reported as mean ± SD, *p < 0.05, **p < 0.01, ***p < 0.001.

We next investigated the distribution of lesion locations in the 45-min MCAO and PT stroke models, using MRI-based morphological reconstructions. Before the stroke intervention, all animals were subject to 4 weeks of food restriction and 3 weeks of daily Staircase training (Figure 2(a)). Video recordings in the Staircase test were taken on day 4 before and days 7, 14, 21 after stroke. MRI measurements were performed on day one after the stroke. Stroke incidence maps were reconstructed according to the Allen Brain Atlas (Figure 2(b)). MCAO and PT resulted in an average lesion volume of 22.99 mm^3^ (±13,2 SD) and 34.03 mm^3^ (±9,8 SD), respectively, with no significant difference for group comparison (Figure 2(c)). Lesions were primarily present in the cortex and striatum, with only minimal lesion volume in other brain areas. MCAO predominantly induced lesions in striatum, with partial cortical involvement (37.75% striatal vs. 16.2% cortical, p < 0.01). In contrast, PT generated small striatal and large cortical lesions (4.97% vs. 46.19%, p < 0.001). Lesion volumes in the remaining brain areas accounted for 2.94% in PT and 3.28% in MCAO. Due to their structural predominance, we used striatal and cortical lesions for further correlation analysis in this study.

### Stroke model specific deficits in the right forepaw following left sided ischemia

**Figure 3.**
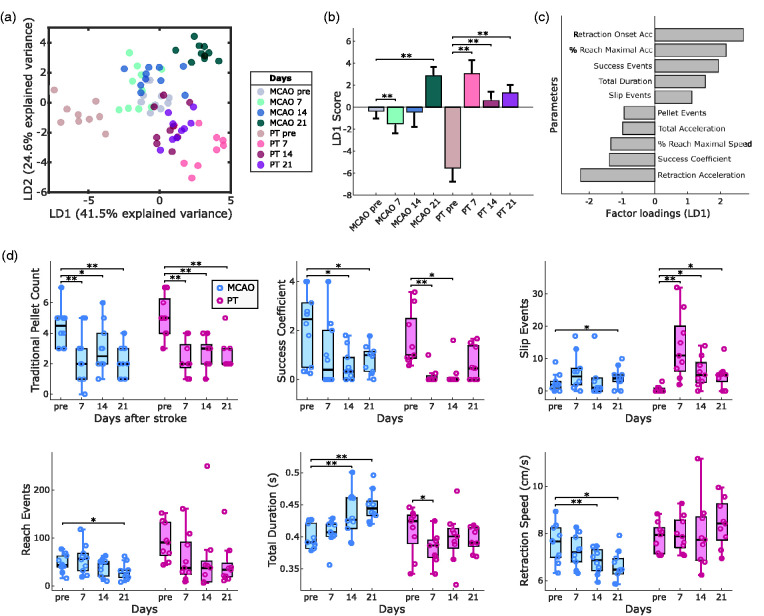
Lesion-specific deficits in the contra-lesional forepaw following MCAO or PT in mice. (a) Linear discriminant analysis (LDA) performed on 30 outcome parameters separates stroke groups and recording days. (b) LD1 scores show significant changes in post-stroke behavior for MCAO and PT groups. (c) The top ten factor loadings on LD1 reveal contribution of speed-related and global parameters to post-stroke deficits. (d) Comparison of post-stroke deficits using traditional quantification (pellet count) vs. refined outcome parameters. Bar graphs are reported as mean ± SD and box plots with Tukey method, *p < 0.05, **p < 0.01.

We next sought to identify the most relevant functional deficits following either MCAO or PT in mice. For this, we computed a set of 30 parameters for motor deficit quantification, including global parameters such as success ratio of reaching attempts, forepaw slip events, or kinematic features of reaching attempts (full parameter list in Table S1). To identify the most relevant features for deficit quantification, we performed a linear discriminant analysis (LDA) for dimensionality reduction based on group information from stroke lesions and recording days (Figure 3 and Fig. S4). The first linear discriminant (LD1) explained 41.5% of the variance in the dataset, and LD2 explained 24.5% (Figure 3(a)). Both stroke groups showed significant changes in LD1 and LD2 scores (p < 0.001). The most prominent differences in LD1 were observed for MCAO on day 21 (p < 0.01) and PT on day 7 (p < 0.01) when compared to pre-stroke values (Figure 3(b) and S3B). Similar effects were observed for LD2 (Fig. S4A). Factor loadings on LD1 revealed that stroke-induced behavioral changes were predominantly related to global motor deficits, movement speed of reach kinematics but not absolute kinematic distances (Figure 3(c)). Together, this multifactorial analysis revealed a predominance of early deficits after PT vs. accumulating late deficits after MCAO.

At the level of individual outcome parameters, traditional pellet count successfully captured post-stroke deficits in both stroke groups, including days 7, 14, and 21 (Figure 3(d)). In contrast, the success coefficient showed significant deficits only on days 14 and 21 after MCAO and on days 7 and 14 after PT. On close inspection, traditional pellet counts tended to overestimate motor abilities in mice before stroke, in case they required a high number of reaches to retrieve individual pellets. For MCAO, we observed a progressive increase in reach duration accompanied by a decrease in forepaw movement speed. Deficits after PT were exemplified by the presence of forepaw slips throughout days 7 to 21 (Fig. S3A). The high number of reaching attempts throughout the experiments confirmed that animals maintain their abilities to perform reaching movements with the arm and preserve high levels of task engagement. In summary, our results identified suitable parameters for the quantification of differential post-stroke motor deficits in the two applied stroke models (Movie S1).

### Correlations of motor deficits with striatal or cortical lesion locations

**Figure 4.**
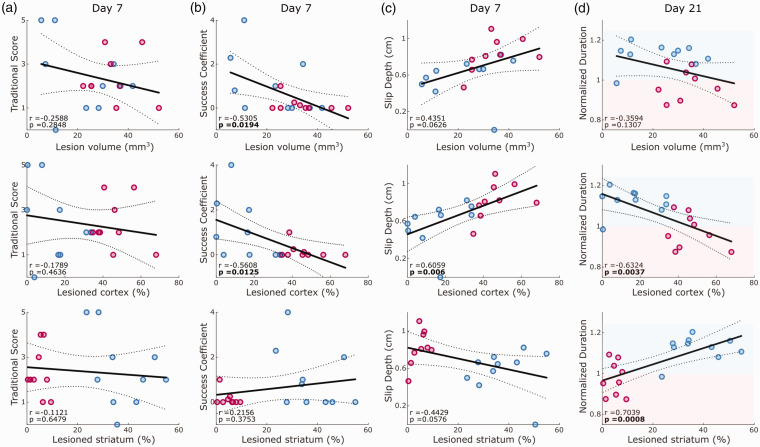
Correlation between outcome parameters and either total lesion volume, cortical or striatal lesion percentage. Correlations are reported for pooled dataset of MCAO and PT animals on day 7 after stroke for traditional pellet count (a), success coefficient (b) and slip depth (c), as well as on day 21 for normalized duration of the reaching cycle (d). Lines indicate linear fit and 95% confidence intervals. Red and blue shaded areas in d indicate decreased or increased reaching duration in comparison to pre-stroke behavior. Values report Pearson coefficients (r) and corresponding p-values. Significant correlations are marked in bold font for p < 0.05.

We next addressed the contribution of lesion locations to the generation of motor deficits. Since both stroke groups showed comparable overall lesion volumes (Figure 2(c)), we pooled the data from both stroke groups for lesion-symptom correlation. Surprisingly, traditional pellet count showed no significant correlations with either total lesion volume or striatal or cortical ischemia (Figure 4(a)). Instead, several other refined outcome parameters revealed lesion-specific correlations (Figures 4(b) to (d) and S5). For example, the success coefficient on day 7 after stroke was significantly correlated with total lesion volume and cortical ischemia (p < 0.05), but not with striatal ischemia (Figure 4(b)). Slip depth highly correlated with cortical ischemia (p < 0.01) but did not reach the significance level for total or striatal lesion volume (Figure 4(c)). The duration of reaching on day 21 was positively correlated with striatal lesion and negatively correlated with cortical lesion size (p < 0.001 and p < 0.01, Figure 4(d)). This finding highlighted the evolution of slow movement following a predominantly striatal ischemia and rapid movement following cortical ischemia. In summary, lesion-symptom correlations provided additional evidence for brain-region-specific deficits following cortical or striatal ischemia in mice.

### Compensatory changes in the ipsi-lesional forepaw after stroke

**Figure 5.**
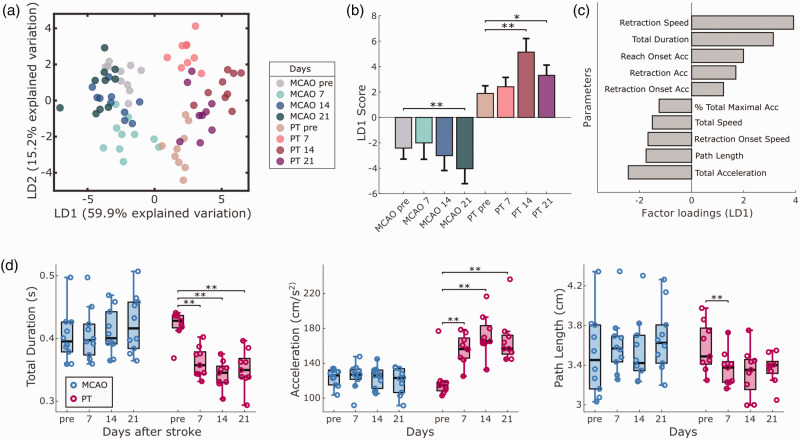
Compensatory kinematic changes in the ipsi-lesional forepaw. (a) Linear discriminant analysis (LDA) identifies the presence of ipsi-lesional motor adaptations, following MCAO and PT. (b) LD1 scores show significant changes on different recording days for both stroke groups. (c) Speed and distance-related parameters primarily account for the observed movement changes, as shown by the top 10 factor loadings on LD1. (d) Individual parameters provide information on new movement strategies after PT, composed of faster and shorter reaching movements. Bar graphs are reported as mean ± SD and box plots with Tukey method, *p < 0.05, **p < 0.01.

We next utilized the bilateral video information to screen for kinematic changes of the ‘non-affected’ ipsi-lesional forepaw in both stroke groups. Using LDA, we discovered changes after PT and MCAO primarily in speed and distance-related kinematic parameters of reaching attempts (Figure 5(a) to (c)). LD1 explained 59.9% of the variance in the dataset, followed by LD2 with 15.2%. Changes in LD1 and LD2 were significant for both stroke groups (p < 0.001), with significant differences in LD1 scores for days 14 and 21 in PT, and day 21 in MCAO animals (Figure 5(b)). Analysis of individual parameters showed significant changes in the PT group for duration, forepaw acceleration, and path length (Figure 5(d)). These movement adaptations after PT occurred when animals dropped in reaching performance not only in the contra-lesional, but also in the ipsi-lesional forepaw on day 7 after stroke (Fig. S2B). Different form the contra-lesional forepaw, the ipsi-lesional forepaw regained pre-lesion levels of reaching success from post-stroke day 14 onwards. Thus, our observations reveal potential compensatory movement strategies of the ipsi-lesional forepaw when reestablishing unilateral reaching success.

### Evaluation of treatment effects of citalopram on post-stroke motor recovery in the PT model

**Figure 6.**
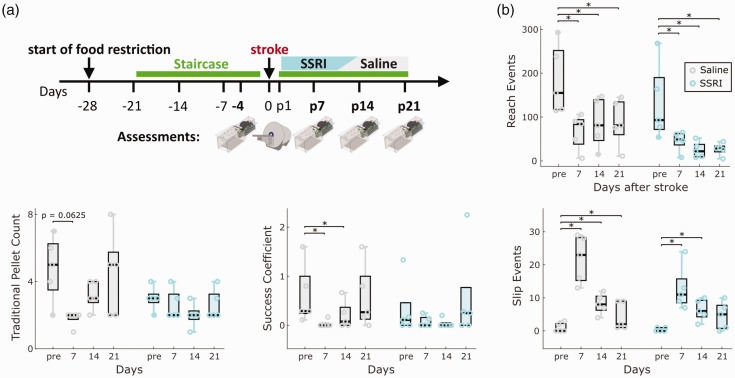
Validating refined outcome parameters during SSRI treatment in the PT model. (a) Experimental design. We compared the effect of daily SSRI treatment with citalopram (10 mg/kg i.p.) to saline injections on the evolution of post-stroke deficits (n = 5 mice per group). Injections started two days after ischemia. Video recordings in the Staircase and MRI measurements were performed on the timepoints indicated in the illustration. (b) Comparison of main outcome parameters, including the number of reach events, traditional pellet count, success coefficient and slip events in their abilities to capture post-stroke deficits and treatment effects. Box plots are reported with Tukey method. *p < 0.05 for post-hoc group comparison with pre-stroke values for each group.

To test the sensitivity of MouseReach in detecting treatment effects, we explored the influence of SSRI on post-stroke motor recovery in an additional set of experiments (Figure 6(a)). We focused our analysis on traditional pellet count, slip events, success coefficient, and reach events (Figure 6(b)). Results showed a significant time effect for all tested parameters (p < 0.05). While reach events exhibited a significant group effect, none of the parameters showed a significant group and time interaction, indicating no confirmed treatment effect of citalopram in our small sample size (n = 5 mice per group). Time-dependent changes in post-stroke deficits were prominent, especially for slip and reach events (p < 0.01) when compared to traditional pellet count or success coefficient (p < 0.05). Post-hoc pairwise comparisons revealed significant deficits for slip and reach events in SSRI and saline groups (Figure 6(b)), but no significant differences in traditional pellet count. This experiment demonstrated that traditional pellet count was influenced by high variance and low learning rates in mice before stroke, while slip events effectively reduced behavioral variance before stroke, resulting in larger effect sizes of post-stroke deficits. These findings validate the reproducibility of identified outcome parameters in detecting post-stroke deficits in the PT model and highlight absence of a strong SSRI treatment effects across multiple outcome parameters.

## Discussion

In the present work, we developed a toolbox for the refined analysis of motor deficits in the Staircase test. As a proof of concept, we show that this toolbox can detect differential motor deficits following either MCAO or PT in mice. Additionally, lesion location in the striatum was linked to progressive symptom manifestation with bradykinesia. In contrast, ischemia in cortex was strongly correlated with forepaw slips from the stabilizing staircase platform. In both stroke models, the number of retrieved pellets was altered, but this traditional measure did not correlate with stroke volume or the amount of cortical or striatal ischemia. Together, these results provide evidence for the benefit of detailed behavioral profiling for translational stroke research.

### Improving lesion-symptom correlations in rodents

Several studies in rodents have shown positive correlations of overall lesion volume with symptom severity in global outcome parameters, such as pellet retrievals. Typically in these studies, the effect of moderately sized lesions was compared to hemispheric infarcts covering most of the striatal and cortical brain tissue.^5,12^ However, large hemispheric strokes may be suboptimal for animal research due to sickness behavior, which may interfere with biological recovery and the accurate evaluation of sensorimotor function.^18^ Under ethical considerations, reducing lesion sizes in rodent models will be of interest for 3 R principles. Moreover, from a translational perspective, most strokes that cause permanent deficits in humans are in the range of small to medium-sized infarcts.^37^ Reducing infarct sizes while maintaining strong correlations of lesion volume and lesion location with symptom severity will aid the quality of translational research. Traditional pellet count may fall short in this endeavor, as for example this parameter could not distinguish between cortical and striatal lesions of comparable size in rats.^12^ Our own results in mice agree with these findings in pointing out limitations of traditional pellet count for lesion-symptom correlation. Instead, identified outcome parameters such as forepaw slips improved the correlation of overall lesion volume and lesion location for moderately sized infarcts in mice.

### Basal ganglia and stroke deficits

In humans, the location of ischemia in either the cortex or basal ganglia leads to differences in motor deficits, outcome prediction, and treatment response.^7–9,11,38,39^ Miyai et al. proposed that the reduced recovery of basal ganglia stroke compared to isolated cortical ischemia is due to distorted communication in cortico-basal ganglia-thalamic networks for motor learning.^38^ Physiologically, this is plausible, as identified structures, such as the dorsolateral striatum, are implicated in motor learning^40^, and stroke recovery in mice is accompanied by the reformation of coordinated neural activity in cortical and striatal ensembles.^41^ Our results show that predominant striatal ischemia after MCAO in mice causes distinct behavioral deficits with an altered time course of symptom manifestation. These insights support the concept that stroke in the basal ganglia should be recognized as a distinct entity for the understanding of recovery and post-stroke therapy.

### Cortex and stroke deficits

A human stroke that affects structures along the corticospinal tract leads to severe hemiparesis.^9,10^ In agreement with previous literature^42^, our results showed that post-stroke deficits in mice after cortical ischemia did not mirror the severity of clinical symptoms in arm movements. These differences have been attributed to the divergence of the functional roles of the corticospinal tract in rodents, non-human primates, and humans.^43,44^ Independently of this discussion, our results establish motor profiles that are unique to mouse models of cortical stroke that may be relevant for translational question on manual dexterity.

### Motor deficits vs. compensatory movements

We interpret the slow increase in reach duration after MCAO, over a period of three weeks, as a progressive motor symptom rather than a compensatory movement. Compensation is present when new motor patterns emerge that improve post-stroke performance, but the original movement does not recover.^16,45,46^ In our MCAO experiments, a decrease in reach duration developed along with declining performance in several outcome measures, including traditional pellet count and success coefficient. Therefore, our analysis can provide a refined view on the distinction between prolonged symptom manifestation and compensation. This capability was further exemplified by progressive changes in movement patterns on the ‘non-affected’, ipsi-lesional forepaw after cortical photothrombosis (PT). Over several weeks, mice after PT progressively reached faster with the ipsi-lesional forepaw and were able to fully recover their ipsi-lesional reaching success to pre-stroke levels. We interpret the faster reaching on the ipsi-lesional forepaw as a possible compensation for the prominent deficits of the contra-lesional forepaw, where forepaw slips indicate an inability to establish a firm grip onto the stabilizing staircase platform. These results also emphasize that Staircase performance requires bilateral coordination, since functional deficits on either side of the body cannot fully be discerned in the test. In experimental cases where the isolation of laterality plays a more central role, the single pellet reaching task may offer a resort strategy. This test only requires gravity support from the ipsilesional forepaw against the floor during contralateral reaching attempts, and – unlike the Staircase test – does not rely on a firm stabilizing ipselesional grip on a platform.

### Limitations of the study

While our study presents a comprehensive analysis of forelimb movements in ischemic stroke models, there are several potential limitations that merit discussion. First, the Staircase test, like other reaching tasks, comes with the drawback of extensive pre-surgical training and some degree of required food restriction. Thereby, the tests introduce confounding factors that need to be controlled for. On the other hand, Staircase or single pellet reaching tests rank among the most sensitive tests to detect forepaw deficits in rodent models of stroke.^18^ Parallel monitoring of multiple animals in the single pellet reaching test could be achieved with similar solutions as presented for our multi-Staircase setup. Yet, a trade-off for the single pellet reaching task will remain the missing ability to assess forepaw slips. Other tests that can evoke forepaw slips, such as the irregular ladder rung, typically address quadrupedal locomotion. Potential trade-offs are, therefore, limited comparability with assessments of arm and hand function in patients.

A second limitation of our current analysis algorithm is that it does not analyze body position with respect to the forepaw during reaching. Body position may influence forepaw trajectories and overall motor coordination. Future iterations of the analysis may therefore benefit from an extension to body and arm tracking. This could also help to distinguish forepaw slips that occur when animals enter the Staircase vs. slips that occur when the animals establishes a firm grip during contralateral reaching.

A third limitation is comparatively small sample sizes in our experiments. The animal numbers were determined based on effect sizes of traditional pellet count from our prior studies. Numbers proved sufficient for addressing our main research questions, yet generalizability to other laboratories will require future validations. In the SSRI experiment, group sizes were planned to reveal therapy effects based on Ng et al., 2015, but we had to exclude more non-learners than anticipated.^47^ While slip events showed significant post-stroke deficits, the group size was insufficient to confirm a statistically significant SSRI treatment effect. Yet, our negative study results on SSRIs in mice align with mixed findings in stroke patients (e.g., FLAME and FOCUS).^48,49^

As a fourth aspect, our results suggest a cortical contribution in the MCAO model for certain deficits, such as slip depth. However, a definitive interpretation of unique striatal and cortical components in the 45-min MCAO model would require larger animal groups, or alternatively addition of models that generate isolated cortical ischemias, such as distal MCAO.

## Conclusion

Our results show that the use of machine learning for tracking forepaw and pellet motions in the Staircase can reveal differential motor deficits in two commonly used mouse models of stroke. The algorithms from this study are freely available for download as a toolbox, *MouseReach*, from online repositories (https://github.com/Wenger-Lab/MouseReach).

## Supplemental Material

sj-pdf-1-jcb-10.1177_0271678X241254718 - Supplemental material for Refined movement analysis in the Staircase test reveals differential motor deficits in mouse models of stroke Supplemental material, sj-pdf-1-jcb-10.1177_0271678X241254718 for Refined movement analysis in the Staircase test reveals differential motor deficits in mouse models of stroke by Matej Skrobot, Rafael De Sa, Josefine Walter, Arend Vogt, Raik Paulat, Janet Lips, Larissa Mosch, Susanne Mueller, Sina Dominiak, Robert Sachdev, Philipp Boehm-Sturm, Ulrich Dirnagl, Matthias Endres, Christoph Harms and Nikolaus Wenger in Journal of Cerebral Blood Flow & Metabolism

sj-mp4-2-jcb-10.1177_0271678X241254718 - Supplemental material for Refined movement analysis in the Staircase test reveals differential motor deficits in mouse models of stroke Supplemental material, sj-mp4-2-jcb-10.1177_0271678X241254718 for Refined movement analysis in the Staircase test reveals differential motor deficits in mouse models of stroke by Matej Skrobot, Rafael De Sa, Josefine Walter, Arend Vogt, Raik Paulat, Janet Lips, Larissa Mosch, Susanne Mueller, Sina Dominiak, Robert Sachdev, Philipp Boehm-Sturm, Ulrich Dirnagl, Matthias Endres, Christoph Harms and Nikolaus Wenger in Journal of Cerebral Blood Flow & Metabolism
